# Guillain-Barré syndrome (GBS) complicated by rhabdomyolysis (RML): Case reports of 2 children and literature review

**DOI:** 10.3389/fped.2022.1001775

**Published:** 2022-10-10

**Authors:** Xin-Ying Yang, Tong-Li Han, Jun-Lan Lv

**Affiliations:** Department of Neurology, Beijing Children's Hospital, Capital Medical University, National Center for Children's Health, Beijing, China

**Keywords:** Guillain-Barré syndrome, rhabdomyolysis, creatine kinase, acute motor axonal neuropathy, acute motor sensory axonal neuropathy

## Abstract

We initially described two children who developed Guillain-Barré syndrome (GBS) complicated by rhabdomyolysis (RML), and reviewed five adult patients from the literature. Through analysis of the clinical features, laboratory examination, treatment and prognostic data from these seven patients, we found that when GBS “meets” RML, the most prominent characteristics were the following: male dominance; limb weakness, pain and respiratory failure could be caused by multiple factors; limb weakness and respiratory muscle paralysis were more serious than with GBS alone; and the probability of mechanical ventilation was increased. Neuroelectrophysiological studies revealed axonal lesions. Close monitoring and timely identification and intervention to remedy potentially fatal complications such as electrolyte disorder multisystem complications and kidney injury are crucial. With plasma exchange, peritoneal dialysis and supportive treatment, the long-term outcome of most patients was satisfactory.

## Introduction

Guillain-Barré syndrome (GBS) is an immune-mediated polyneuropathy with 100,000 new cases worldwide every year. It is the most common cause of acute flaccid paralysis. The molecular mimicry between pathogenic microorganisms and nerve antigens is the main driving force of the disease (1). The classic symptoms of GBS are rapidly progressive bilateral limb weakness with or without cranial nerve paralysis and autonomic nerve dysfunction. Some patients initially experience muscle pain or nerve root pain (2). During the course of GBS, 16–27% of patients show an elevation of serum creatine kinase (CK) levels (3, 4) but only rarely is GBS complicated with rhabdomyolysis (RML) (5–9). The research history of GBS in Beijing Children's Hospital (BCH), which is affiliated with Capital Medical University, can be traced back to the 1990s. Important contributions were the discovery that the main clinical subtype of GBS in China was acute motor axonal neuropathy (AMAN), and Campylobacter jejuni was the most common associated infectious pathogen (10). Here, we report two childhood patients who developed GBS complicated by marked RML, and review the literature to heighten awareness of the clinical manifestations of these combined disorders, as well as emphasize the diagnostic procedures, and their treatments and outcomes. All studies were approved by the appropriate ethics committee of BCH.

## Case presentation

### Patient 1

A 3-year-old boy was hospitalized on March 29, 2016. The chief complaint was “limb weakness for 2 days”. Ten days before symptom onset, he was vaccinated with “epidemic meningitis vaccine”. Two days prior to admission, the child could only walk slowly, but was hardly able to squat or carry moderately heavy loads in his hands. The symptoms progressed, and the day prior to admission, he could not stand alone or lift his arms, his cough and chewing were weak, and his voice was hoarse and his legs were painful. On the day of admission, he could not turn over or raise his head. His admission vital signs included a temperature of 37°C, pulse of 119 beats/min, respiratory rate of 22/min, and blood pressure of 95/46 mmHg. His mental status was clear, but his voice was markedly reduced, head elevation was unstable, neck rotation and shrugging were weak, the posterior drip sign was positive. The pharyngeal reflex was decreased and the deep tendon reflexes of the limbs were absent. Proximal limb muscle strength was graded III, distal was graded II, and the straight leg lifting test was positive. Dermatographism of the limbs and trunk was positive.

Admission laboratory tests that were normal included whole blood cell analysis; routine urine and stool testing; blood electrolyte, liver and kidney function tests, myocardial enzyme spectrum, and coagulation function. There were no positive findings for antinuclear antibodies, anti-double stranded DNA antibodies, thyroid function, poison screening, urine gas chromatography-mass spectroscopy and blood tandem mass spectrometry analysis. Aetiological screening included serum TORCH IgM/IgG antibodies; EBV-CA-IgG, EBV-CA-IgM, EBV-EA-IgA, and EBV-NA-IgG antibodies; and Mycoplasma antibodies, which were all negative. There were no abnormalities on a cranial CT (Table 1).

**Table 1:** Clinical manifestation, laboratory examination, treatment and outcome of patients.

		**BCH Case1**	**BCH Case2**
Clinical manifestation (course of disease)	Sex	Male	Male
	Age	3-y	12-y
	Prodromic event	Meningococcal vaccination	Diarrhea
	Precursor infection pathogen	None	Unknown
	First symptom	Limb weakness	Limb weakness
	HG score at nadir	4 (d4)	5 (d8)
	Pain	Nerve root pain (d2) muscle tenderness (d3)	Numbness (d5) nerve root pain (d6)
	Cranial nerve paralysis	V${}_{{}^{\prime }}$VII${}_{{}^{\prime }}$IX${}_{{}^{\prime }}$X${}_{{}^{\prime }}$XI	III${}_{{}^{\prime }}$IV${}_{{}^{\prime }}$V${}_{{}^{\prime }}$VI${}_{{}^{\prime }}$VII${}_{{}^{\prime }}$IX${}_{{}^{\prime }}$X${}_{{}^{\prime }}$XI
	Respiratory muscle paralysis	I° (d5)	III° (d8)
	Respiratory failure	None	Type II
	Acute kidney injury	None	Yes
	Others	Skin scratch sign	Anhidrosis, skin dryness, tachycardia,hypotension
Laboratory examination (course of disease)	CK elevation initiation/peak/recovery	d5/d9/d20	d11/d12/d21
	CK Max (iu/L)	7,908	17,840
	MB elevation initiation/peak/recovery	d5/d5/d20	d11/d11/d21
	MB Max (ng/ml)	>1,200	491
	Bun Cr	Normal	↑
	Blood electrolyte	Normal	Normal
	Blood gas analysis	Normal	PCO2↑
	CSF albuminocytologic dissociation	Yes (d7)	Yes (d15)
	Serum antiganglioside antibody	Untest	Anti GM1-IgG and anti GD1b-IgG (+)
	Neurophysiologic studies	AMAN (d21)	AMSAN (d14)
	MRI of cranial and spinal cord	Normal	Normal
Treatment (course of disease)	IVIG	2 g/kg 2 times	2 g/kg
	Glucocorticoid	Methylprednisolone	Not used
	PE (initial/ending)	Not used	6 times (d10/d17)
	Mechanical ventilation (initial/ending)	Not used	d8/d19
	Alkalized therapy (initial/ending)	d5–d18	d11–d20
Outcome (course of disease)	Initial recovery	d20	d15
	HG score (1 month)	4	4
	HG score (3 month)	3	3
	HG score (6 month)	0	0

BCH, Beijing Children's Hospital; CK, creatine kinase; MB, myoglobin; Bun, blood urea nitrogen; Cr, creatinine; AMAN, acute motor axonal neuropathy; AMSAN, acute motor sensory axonal neuropathy; IVIG, intravenous immunoglobin; PE, plasma exchange; HG score, Hughes score.

**Figure 1:**
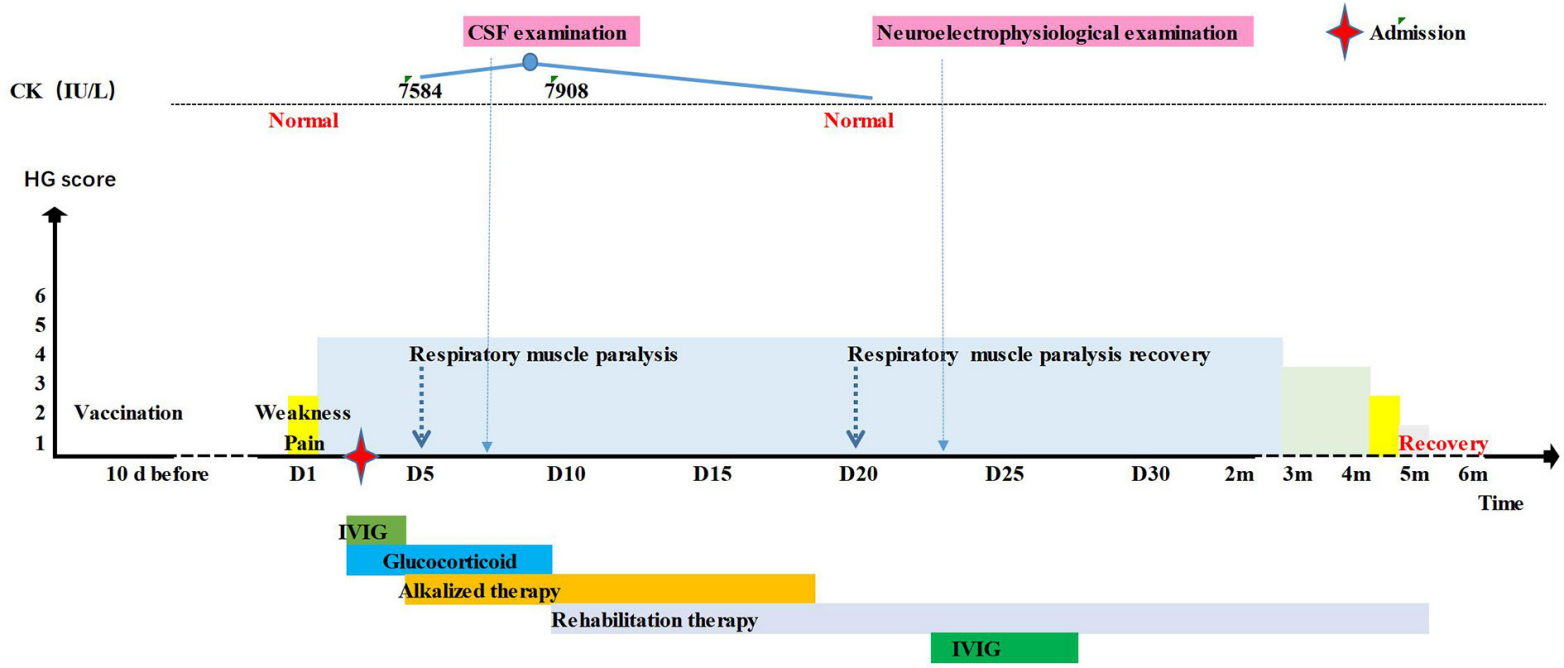
Timeline of the patient 1's clinical manifestations, laboratory examinations and treatments.

With a tentative working diagnosis of GBS, the patient received intravenous immunoglobulin (IVIG) (2 g/kg) for 2 d. To alleviate his obvious nerve root pain, methylprednisolone 2 mg/kg/d was given to improve the inflammatory oedema of the nerve root. On the third day following admission, the patient's condition worsened: dysphagia was aggravated, the pharyngeal reflex disappeared, the muscle strength of proximal and distal extremities were reduced to grade II and grade I respectively, and he complained of muscle pain. The blood enzyme spectrum was elevated: CK 7584 U/L (25–200 U/L), creatine kinase isoenzymes-MB (CK-MB) 213 U/L (25–200 U/L), aspartate aminotransferase (AST) 388 U/L (14-44 U/L), alanine transaminase (ALT) 261.2 U/L (7–30 U/L), and serum myoglobin (MB) >1,200 ng/ml (0–140 ng/ml). However blood urea nitrogen (BUN), creatinine (CR) and routine urine tests were normal. Examination of the cerebrospinal fluid (CSF) showed albuminocytologic dissociation (WBC counts was 0, protein was 583 mg/L) (20–450 mg/L). Cranial and spinal cord MRI was normal. The patient was rehydrated and administrated alkalization treatment. The CK peaked at 7908 IU/L, CK-MB reached 237 IU/L, then decreased gradually and reached normal on the 18th day. On the 19th day, neurophysiologic studies showed motor axonal lesions. On the 21st day, he was given IVIG 2g/kg. On the 26th day, the child could turn over, the pain disappeared and he was discharged for rehabilitation. One month later, he could sit alone, and two months later, he could walk alone. Finally, he returned to normal after 4 months (Figure 1).

The final diagnosis: (1) Guillain-Barré syndrome (acute motor axonal neuropathy, AMAN), complicated with V, VII, IX, X and XI cranial nerve paralysis; (2) I° respiratory muscle paralysis; and (3) rhabdomyolysis.

### Patient 2

A 12-year-old boy was hospitalized on February 24, 2018, with a chief complaint of “limb weakness for 6 days and sensory impairment for 1 day”. Two days prior to symptom onset, the boy experienced diarrhea. Six days before admission, the patient squatted and climbed stairs with difficulty and could not carry heavy objects with his hands. Three days before admission, his voice decreased, and he complained of visual ghosting. One day before admission, he felt numbness in his hands and feet. On the day of admission, he could not walk and had difficulty chewing and swallowing. Admission vital signs included temperature of 36°C, respiratory of 16/min, pulse of 94 beats/min, and blood pressure of 120/80 mmHg. His mental status was clear, but he could not stand or sit. He had bilateral ptosis, limited upper vision and abduction. Mastication, neck rotation and shrugging were weak and the pharyngeal reflex was decreased. Hypotonia was observed in all four limbs, and the deep tendon reflex was absent. The proximal muscle strength of the limbs was grade III, and the distal muscle strength was grade II. Limb numbness and other sensation examinations were normal. The straight leg lift test was positive.

**Figure 2:**
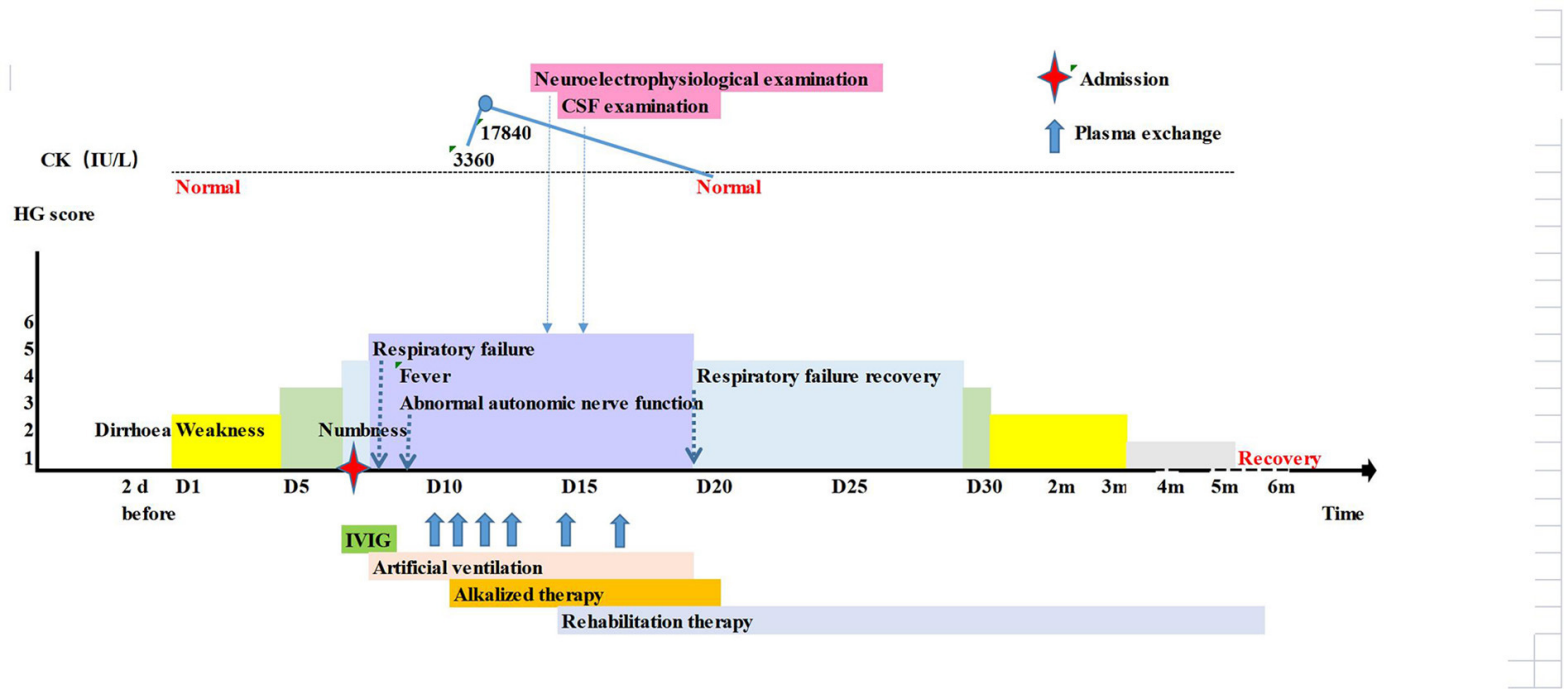
Timeline of the patient 2's clinical manifestations, laboratory examinations and treatments.

Normal laboratory tests included: the whole blood cell analysis, routine and stool examinations, blood electrolyte, liver and kidney function, muscle zymogram spectrum, and coagulation function. Urine gas chromatography-mass spectroscopy was in the normal range. The IgM and IgG antibodies against TORCH, Coxsackie, ECHO and EB virus were negative. No abnormalities were found on cranial CT (Table 1). Because GBS was considered, the patient received IVIG therapy (25 g/d). Nevertheless, the next day, the child's condition progressed, and he exhibited dyspnoea, right peripheral facial paralysis, disappearance of the pharyngeal reflex, III° respiratory muscle paralysis and type II respiratory failure. Therefore, he was intubated and mechanically ventilated. On the third day, the child's condition had not improve and he developed a fever of 40.2°C and abnormal autonomic nerve function including anhidrosis, skin dryness, tachycardia and hypotension. Plasma exchange (PE) was performed from the 4th day, initially once a day times 4, then once every other day times 2. On the 5th day, the blood CK increased to 3360 U/L (25–200 U/L). The following day, the CK rose dramatically to 17840 U/L (25–200 U/L), with additional abnormal tests including ALT 97.5 U/L (7–30 U/L), MB 491.2 ng/mL (0-140 ng/mL), BUN 11.23 mmol/L (2.5–6.5 mmol/L), and Cr 169.6 μmol/L (19–44 μmol/L). The patient was treated with rehydration and alkalinization. Neurophysiological studies showed axonal lesions of motor and sensory nerves, but the cranial and spinal MRI were normal. On the 9th day, the CSF test showed albuminocytologic dissociation with protein elevation of 1,858 mg/L and a normal WBC count, and the serum anti-GM1 IgG antibody and anti-GD1b IgG antibody were positive. Starting on the 9th day, the patient's condition improved gradually. On the 13th day, the patient underwent tracheotomy and artificial nasal ventilation. His muscle strength improved to grade IV, and the autonomic nerve dysfunction resolved. The CK returned to normal on the 15th day, and kidney function returned to normal on the 17th day. One month later, the boy could walk alone, his cranial nerve examination was normal. Five months later, he had full recovery of movement and sensation (Figure 2).

Final diagnosis: (1) Guillain-Barré syndrome (acute motor sensory axonal neuropathy, AMSAN), complicated with III, IV, V, VI, VII, IX, X and XI cranial nerve paralysis; (2) III° respiratory muscle paralysis; (3) Type II respiratory failure; (4) rhabdomyolysis; (5) pneumonia.

## Discussion

There are various causes of rhabdomyolysis including trauma, burns, vigorous exercise, drugs, toxins, or infections such as viral myositis. The clinical triad includes muscle weakness, muscle pain and myoglobinuria manifesting as dark red or brown urine. Clinically, rhabdomyolysis can range from asymptomatic to severely critical states such as volume depletion, marked metabolic and electrolyte disorders and acute kidney injury (11). At present, the widely used diagnostic standard is CK > 1,000 IU/L (12).

The two young patients in our department manifested acute onset, progressive and symmetrical limb weakness, cranial nerve paralysis, accompanied by sensory abnormalities and autonomic nerve dysfunction. Respiratory muscle paralysis and respiratory failure occurred in the course of the disease. The CSF test demonstrated albuminocytologic dissociation, and neurophysiological studies showed axonal lesions. Their function nadir occurred at 1–2 weeks, but they ultimately fully recovered and have not experienced any sequelae with follow-up of 6 and 4 years. These data confirmed the diagnosis of GBS and with marked CK increases on the 5th and 11th days with peaks of 7,908 IU/L and 17,840 IU/L, respectively, both were diagnosed with rhabdomyolysis.

Review of the medical literature identified 5 patient case reports of GBS that were complicated with RML (Table 2) (5–9). Therefore, a total of 7 GBS patients experienced concomitant RML. Interesting characteristics of this group included a ratio of male to female of 6:1, suggesting male dominance. Five patients experienced precursor events, including vaccination in one and infection in four. The latter included 2 cases of diarrhea, 1 case of fever and 1 case of pneumonia. Two studies that focused on the marked elevation of creatine kinase in GBS suggested that male sex and prodromal infection were more common in this group (3, 4), consistent with the data from these 7 patients.

**Table 2:** Literature review and patient data.

		**Case 1 (5)**	**Case 2 (6)**	**Case 3 (7)**	**Case 4 (8)**	**Case 5 (9)**
Clinical manifestation (course of disease)	Sex	Male	Male	Male	Female	Male
	Age	25 y	54 y	24 y	25 y	21 y
	Prodromic event	Not mentioned	Not mentioned	fever	Pneumonia	Diarrhea
	Precursor infection pathogen	Unknown	Unknown	Unknown	Mycoplasma pneumoniae	Campylobacter jejuni
	First symptom	Weakness and pain	Weakness and pain	Limb weakness	Limb weakness	Limb weakness
	HG score at nadir	4	5	6	5	4
	Pain	Transient chest and upper extremities pain	Lower limb pain	Not mentioned	Not mentioned	Lower extremity and back pain,Limb muscle spasm and severe pain
	Respiratory muscle paralysis	None	Yes	Yes	Yes	None
	Respiratory failure	None	Yes	Yes	Yes	None
	Acute Kidney injury	Yes	Unknown	Yes	Yes	None
	Others	Urinary retention	Urinary retention		Anuria, hypertension, pleural effusion	
Laboratory examination (course of disease	CK max (iu/L)	10,150	1,134	7,002	77,700	1,917
	CK elevation initiation /recovery	d1/d21	d1/not mention	d20/?	d1/not mentioned	d39/d100
	MB (ng/ml)	Not mentioned	Not mentioned	>1,000	Not mentioned	580
	Others			K↑P↑Ca↓/ metabolic acidosis.		
	CSF albuminocytologic dissociation	No (d1/d14)	Not mentioned	Yes (d20)	Not mentioned	y es (not mentioned)
	Serum antiganglioside antibody	Not mentioned	Negative	Negative	Not mentioned	GM1-IgM GD1b-IgM
	Neurophysiologic studies	AMSAN (d14)	AIDP (d3)	AMSAN (d20)	AMSAN (d7)	AMAN (not mentioned)
	Muscle biopsy	Necrosis, edema (not mentioned)	Not done	normal (d23)	Normal (not mentioned)	n ot done
Treatment and outcome (course of disease	IVIG	Not used	Not used	Not used	Not mentioned	Yes
	PE/PD	PE	PE	PD	PD	PE
	Mechanical ventilation	Not used	Yes (d3-d12)	Yes (d23)	Yes	Not used
	Outcome	3 m improved; 10 m walk alone	d45 muscle strength grade IV	d24 died of cardiac arrest	d68 Spontaneous breathing 5m walk alone	3 m pain and weakness recovery

CK, creatine kinase; MB, myoglobin; K, blood potassium; P, blood phosphorus; Ca, blood calcium; AMSAN, acute motor sensory axonal neuropathy; AIDP, acute inflammatory demyelinating polyneuropathies; AMAN, acute motor axonal neuropathy; IVIG, intravenous immunoglobin; PE, plasma exchange; PD, peritoneal dialysis.

### Limb weakness and respiratory muscle weakness

The main symptom of GBS is symmetrical progressive limb weakness. In approximately 1/3 of the patients, the muscle disabilities are mild to moderate (HG < 3 points) and the ability to walk throughout the course of the disease is maintained (13). In the present series of 7 patients, all were nonambulatory, with an HG > 4 points. Recognizing that one of the RML triad is weakness, it was difficult to distinguish whether it was involved in these patient's dyskinesia. Two patients in our center and cases 3 and 5 from the literature review were unable to walk before the CK became elevated, suggesting that the motor disability was attributable to GBS. Approximately 25% of GBS patients develop respiratory failure that requires mechanical ventilation (13). Of these 7 patients, 5 (71.5%) suffered respiratory muscle paralysis, and 4 (57%) required mechanical ventilation. This suggests that the severity of limb and respiratory muscle weakness in GBS may have been intensified by the RML.

Two studies suggested that the increase in CK was not related to GBS (3, 4). With the severity of motor nerve dysfunction in the present series of 7 patients where GBS “meets” RML, the combination should be considered an early warning indicator for mechanical ventilation.

### Pain

Approximately 1/3 of GBS patients experience severe pain during the course of the disease, which may be neurogenic or myogenic (14). Notably, one of the rhabdomyolysis symptom triad is muscle pain. Obviously, muscle pain is a non-specific symptom, and it is difficult to distinguish which of the two disorders was principally responsible for these patient's pain. The two patients in our department mainly had limb traction pain, and 1 case was accompanied by muscle tenderness, which occurred earlier than CK elevation. GBS may be the main cause of pain, and RML may also be a factor.

In the literature review, 2 case reports described the pain in detail. Case 1 reported transient chest and upper limb pain, which was considered to possibly be neurogenic pain. Case 5 complained of back pain in the early stage, and severe muscle spasm pain was present in the later stage, followed by an increase in CK. This pain may have been due to rapid and extensive denervation caused by severe axonal degeneration of motor nerve terminals. Local muscle exhibits high excitability, leading to repeated muscle spasm and CK release. When GBS patients complain of pain, it is necessary to carefully identify its nature. Particularly when muscle tenderness is present, rhabdomyolysis should be strongly suspected.

### Acute kidney injury (AKI)

AKI is the most serious complication of RML. The mechanism of RML involves muscle cell lysis with strong likelihood of blood electrolyte disorders, including hyperkalaemia, hyperphosphatemia, hyperuricaemia, hyperanionic interstitial metabolic acidosis and hypermagnesemia, accentuated when renal failure is present (15). The incidence of AKI in children with RML is approximately 5–35% (16–18). GBS is rarely complicated by renal function impairment. Of the seven patients in this series, AKI developed in 4 cases. When GBS patients develop AKI, prompt and careful investigation for the cause should be undertaken, paying particular attention to RML. Other factors include underlying disease, precursor infection or IVIG-related renal function injury.

### GBS subtype

The correlation between elevation of creatine kinase due to RML and GBS are classified as axonal lesion subtype (AMAN or AMAN with RCF) (3, 4). This conclusion also supports the hypothetical mechanisms of GBS with elevated creatine kinase, in which denervation of the muscle leads to muscle enzyme release. Our study included 7 patients. Only one electrophysiological examination of a 54-year-old male showed AIDP changes, but this test was completed on the third day of his disease course. It is impossible to verify whether the follow-up review confirmed axonal lesions with RCF.

### Differential diagnosis and muscle biopsy

The differential diagnosis of GBS complicated by rhabdomyolysis should be distinguished from critical illness polyneuropathy and critical illness myopathy (CIPNM) and potential genetic or metabolic diseases. CIPNM is a complication arising after the onset of critical illness, such as sepsis, systemic inflammatory response syndrome (SIRS) and multiple organ failure (19). This patient group suffered from limb weakness at onset but not other primary severe diseases, CIPNM diagnosis was insufficient. However, it is difficult to distinguish whether CIPNM occurred due to muscle microcirculation disturbance caused by limb compression and immobility in the early stage of GBS, and whether CIPNM participated in part of the disease process. In clinical practice, when necessary, muscle biopsy and gene testing may help to make the diagnosis. In the reviewed cases, three patients underwent muscle biopsy, one showed muscle tissue necrosis and edema, and the other two cases were normal. It should be noted that in the early stage of RML, a muscle biopsy may be normal or reveal nonspecific signs except necrosis. When rhabdomyolysis persists and the etiology is unclear, muscle biopsy is an optional method to find clues. If the condition permits, it is recommended to take a biopsy several weeks or months after the occurrence of clinical symptoms to improve the positive rate. After treatment, the conditions of two young patients in our department improved and CK decreased to normal, so muscle biopsy was not performed. If metabolic myopathy is clinically suspected, a muscle biopsy should be performed as soon as possible.

### Treatment and outcome

GBS is a self-limiting disease, and supportive treatment with prevention or remedy of secondary complications in the acute phases are the determinant of prognosis. In the process of recovery, prolonged bed rest, limb immobilization and compression, respiratory dysfunction and severe infection lead to muscle microcirculation injury. Conversely, RML and its complications also bring challenges to the treatment of GBS. Plasma exchange (PE) or peritoneal dialysis (PD) may become necessary and are highly effective. One patient received PE treatment in our center. In the literature review, 3 patients received PE, and 2 patients received PD. One patient died of cardiac arrest resulting from hyperkalemia, the other patients had satisfactory recoveries.

## Conclusions

When GBS “meets” RML, limb weakness, pain and respiratory failure may be caused by multiple factors, limb weakness and respiratory muscle paralysis are more serious than with GBS alone, and the probability of mechanical ventilation is increased. Neuroelectrophysiological studies often reveal axonal lesions. With close monitoring and timely identification of potentially fatal complications such as electrolyte disorders, multisystem complications and kidney injury, and actively adopting PE, PD and other supportive treatment, the long-term outcome of most patients was satisfactory.

## Data availability statement

The original contributions presented in the study are included in the article/supplementary material, further inquiries can be directed to the corresponding author.

## Author contributions

All authors listed have made a substantial, direct, and intellectual contribution to the work and approved it for publication.

## Conflict of interest

The authors declare that the research was conducted in the absence of any commercial or financial relationships that could be construed as a potential conflict of interest.

## Publisher's note

All claims expressed in this article are solely those of the authors and do not necessarily represent those of their affiliated organizations, or those of the publisher, the editors and the reviewers. Any product that may be evaluated in this article, or claim that may be made by its manufacturer, is not guaranteed or endorsed by the publisher.
